# Reducing asthma attacks: consider patients' beliefs

**DOI:** 10.1038/npjpcrm.2015.21

**Published:** 2015-04-02

**Authors:** Anders Østrem, Rob Horne

**Affiliations:** 1 Gransdalen Legesenter, Gransdalen 29, Oslo, Norway; 2 UCL School of Pharmacy, University College London, London, UK

Dear Sirs,

The editorial by Mike Thomas and Eric Bateman^1^ focusses on a very important, clinical challenge—how to reduce asthma attacks. One of the issues discussed is the apparent lack of the patient's understanding of the information regarding self-management, which can lead to non-adherence. We agree with the authors that ‘persuading some patients that they need to take regular ICS (even as a combination inhaler) is an ongoing challenge’. We find it timely to emphasise the knowledge we have regarding how we can improve asthma control by understanding the patient’s perspective.

Patients do not blindly follow the treatment advice but are strongly influenced by their ‘common-sense’ beliefs about their illness and treatment.^2,3^ Even if the advice comes from a trusted health-care worker the patient will evaluate whether it makes sense in the light of their understanding and beliefs. Adherence to medication is especially influenced by the patients’ beliefs about the prescribed medication, particularly how they judge their personal need for it relative to their concerns about the potential adverse consequences of taking it. A recent meta-analysis showed that this simple Necessity Concerns Framework was helpful in explaining non-adherence across 94 peer-reviewed publications from 18 countries involving over 25,000 patients, across 24 long-term conditions including asthma.^4^ Many patients with asthma doubt their personal need for daily doses of ICS or have concerns about them, even when they experience no ‘side-effects’.^5–7^

Doubts about the necessity of ICS often arise from the patient's beliefs about asthma.^7^ In order to perceive that we need treatment we have to see a close fit between our understanding of the problem (the illness) and the proposed solution (the treatment). Many patients with asthma simply don’t see a good fit. The medical model of asthma as a chronic condition that requires daily preventative medication may be at odds with their experience of asthma as an episodic condition in which symptoms come and go. Daily ICS may not make sense to them if their belief is ‘no symptoms, no asthma.’^8^ One could suspect that in many of the patients included in the linked paper by Patel *et al.*,^9^ the extreme overuse of SABA (short-acting β2-agonist) could be explained by the patient’s own beliefs about asthma and how best it can be treated.

The challenge for the health-care worker, be it the GP, practice nurse, pharmacist or hospital specialist, is to understand the patient’s beliefs about asthma and its treatment. This is the starting point for tailoring the prescription and providing support to meet the needs of the individual. A three-point perceptions and practicalities approach^2^ might be a good start to tailor the support to enhance the patient’s motivation and ability to get the best from the appropriate treatment:

1. Present a ‘common-sense’ rationale or ‘story’ explaining why daily treatment is necessary, even in the absence of symptoms.
2. Elicit and address the concerns about medication.
3. Make the regimen as easy and convenient as possible to check that the patient is able to use the treatment (including inhaler technique where appropriate).

We agree with the authors of the editorial that we still have a long way to go before optimal, effective self-management is achieved, but we suggest that an understanding of the patient’s perspective about asthma and its treatment offers the gateway to this.
